# Low Dose Nicotine and Antagonism of β2 Subunit Containing Nicotinic Acetylcholine Receptors Have Similar Effects on Affective Behavior in Mice

**DOI:** 10.1371/journal.pone.0048665

**Published:** 2012-11-07

**Authors:** Shawn M. Anderson, Darlene H. Brunzell

**Affiliations:** 1 Department of Pharmacology and Toxicology, Virginia Commonwealth University School of Medicine, Richmond, Virginia, United States of America; 2 Interdepartmental Neuroscience Graduate Program, and Institute for Drug and Alcohol Studies, Virginia Commonwealth University School of Medicine, Richmond, Virginia, United States of America; Neuroscience Campus Amsterdam, VU University, The Netherlands

## Abstract

Nicotine leads to both activation and desensitization (inactivation) of nicotinic acetylcholine receptors (nAChRs). This study tested the hypothesis that nicotine and a selective antagonist of β2*nAChRs would have similar effects on affective behavior. Adult C57BL/6J male mice were tested in a conditioned emotional response (CER) assay which evaluates the ability of an aversive stimulus to inhibit goal-directed behavior. Mice lever-pressed for a saccharin reinforcer according to a variable schedule of reinforcement during sessions in which two presentations of a compound light/tone conditioned stimulus (CS) co-terminated with a 0.1 or 0.3 mA, 0.5 s footshock unconditioned stimulus (US). During testing in the absence of the US, mice received doses of i.p. nicotine (0, 0.0032, 0.01, 0.032, 0.1 mg/kg) or a selective β2 subunit containing nAChR (β2*nAChR) antagonist dihydro-beta-erythroidine (0, 0.1, 0.3, 1.0, 3.0 mg/kg DHβE). There was a dose-dependent effect of nicotine revealing that only low doses (0.01, 0.032 mg/kg) increased CER suppression ratios (SR) in these mice. DHβE also dose-dependently increased SR at the 3 mg/kg dose. In ethological measures of fear−/anxiety-like behavior, these doses of nicotine and DHβE significantly reduced digging behavior in a marble burying task and 0.3 mg/kg DHβE promoted open-arm activity in the elevated plus maze. Doses of nicotine and DHβE that altered affective behavior had no effect on locomotor activity. Similar to previous reports with anxiolytic drugs, low dose nicotine and DHβE reversed SR in a CER assay, decreased digging in a marble burying assay and increased open arm activity in the elevated plus maze. This study provides evidence that inactivation of β2*nAChRs reduces fear-like and anxiety-like behavior in rodents and suggests that smokers may be motivated to smoke in part to desensitize their β2*nAChRs. These data further identify β2*nAChR antagonism as a potential therapeutic strategy for relief of negative affect and anxiety.

## Introduction

Human and animal studies indicate that nicotine exerts its psychoactive effects by binding to nicotinic acetylcholine receptors (nAChRs) in the brain [1], [2]. nAChRs comprised of the β2 subunit (β2*nAChRs; *denotes assembly with other subunits) have high binding affinity for nicotine and the endogenous neurotransmitter, acetylcholine (ACh) [3]–[6]. β2*nAChRs are enriched on neurons in limbic system brain areas that regulate both affect and reward [3], [6]–[16] suggesting that these nAChR subtypes may serve a dual role in supporting reward-like behavior and relieving negative affect. nAChRs are ion channels that can be activated as well as desensitized (inactivated) by nicotine [17]–[20]. A preponderance of the evidence suggests that activation of β2*nAChRs supports nicotine conditioned place preference and nicotine self-administration, models of nicotine reward and reinforcement [21]–[32] (but see [33]). These studies used a conditioned emotional response (CER) assay, a marble burying task and an elevated plus maze experiment to test the hypothesis that inactivation of β2*nAChRs attenuates fear and anxiety-like behavior in mice.

CER is an appetitive, operant task in which lever pressing maintained by a positive reinforcer (saccharin solution) is interrupted by presentation of a conditioned stimulus (CS, light and tone) that co-terminates with an aversive unconditioned stimulus (US, 0.1 or 0.3 mA, 0.5 s mild footshock). This study tested if nicotine and a selective antagonist of β2*nAChRs, dihydro-beta-erythroidine (DHβE), would attenuate conditioned suppression of responding in the presence of an aversive CS in the absence of footshock. During CER, subjects serve as their own controls within sessions to return a suppression ratio (SR) score  =  A/(A+B) where A is lever pressing during the 60 s CS period and B is lever pressing during the 60 s prior to CS presentation (Pre-CS). An SR ≈ 0 is indicative of conditioned suppression whereas SR ≈ 0.5 indicates that rodent responding is unaffected by presentation of the CS. The CER assay has good face validity for tobacco users who experience stressors during goal-oriented behavior on a daily basis. Separate groups of mice were tested using an ethological marble burying task where increased digging, rather than suppression of activity, is thought to be interpretive of negative affective behavior, and in an elevated plus maze assay where increased activity in the open arms, relative to the enclosed arms of the maze, is thought to reflect anxiolysis-like behavior. As a follow-up to experiments that evaluated affective-like phenotype, a locomotor activity assay using a beam-break apparatus confirmed that doses of nicotine and DHβE in these experiments did not affect locomotor activity.

## Materials and Methods

### Subjects

Twenty nine C57BL/6J, adult, male mice from Jackson Labs (Bar Harbor, ME) or derived in a Virginia Commonwealth University (VCU) breeding colony were used in these studies. Mice had *ad libitum* access to both food and water in their home cages. Animals were group-housed (2–5 per cage) with 1/8 inch corn cob bedding in a vivarium with a 12 h light/dark cycle (lights on 0600). Mice were habituated to the test room and experimenter handling for 3 days prior to any training or testing.

### Ethics Statement

Efforts were made to minimize mouse discomfort in these experiments. Mild footshock without analgesia and experimenter injections were necessary to perform these studies that model affective-like behavior in mice. Experiments were approved by the VCU Institutional Animal Care and Use Committee (Protocol Number: AM-10163) and were in compliance with the *Guide for Care and Use of Laboratory Animals* (Institute of Laboratory Animal Resources, 2010).

### Apparatus

CER experiments were conducted in mouse operant chambers (21.6 cm×17.8 cm×12.7 cm; Med Associates, St. Albans, VT). An LED cue light with an opaque cover was positioned 5.5 cm above the operant lever with a liquid dipper receptacle centered on the same wall. A speaker and a 2.24 watt incandescent house-light were positioned 9.5 cm from the floor on the opposite wall of the operant chamber. The floor consisted of steel rods (0.32 cm in diameter placed 0.79 cm apart) connected to a Med Associates shock generator/scrambler. All data were collected via Med PC software. Marble burying took place in a polycarbonate cage (33 cm×21 cm×9 cm high) filled with 5 cm of loose wood chip bedding (Harlan Sani-Chip, Indianapolis, IN). The elevated plus maze was constructed of wood with white laminated flooring on two (5×30 cm) open arms that were perpendicular to two equivalent, white, laminated, enclosed arms with 15.25 cm black Plexiglass wall enclosures. The entire apparatus was elevated 68 cm above the floor. Experimentation took place under fluorescent light illumination. A ceiling-mounted camera was interfaced to a PC for collection of data using ANY-maze tracking software by Stoelting (Wood Dale, IL). Locomotor testing was conducted in two adjoining chambers (measuring 26.5 cm×12.7 cm×26.2 cm and 16.8 cm×12.7 cm×12.7 cm). A locomotor unit was defined as the breaking of two adjacent light beams (3 cm apart). Illumination was provided by a single 23 watt fluorescent light bulb. Data was collected using Med Associates software. All experimental chambers were cleaned between animals with 2% Nolvosan (Pfizer Animal Health, Madison, NJ).

### Drugs

Nicotine hydrogen tartrate (Sigma Aldrich, St. Louis, MO) was dissolved in 0.9% sterile saline with pH adjusted to 7.1–7.3. DHβE (Sigma Aldrich, St. Louis, MO) was dissolved in 0.9% sterile saline. Nicotine doses are expressed as freebase and DHβE doses are expressed as hydrogen bromide salt. As with previous nicotine place conditioning studies [28], [34], injections were delivered i.p. in volumes of 0.1 ml/30 g. Nicotine was administered immediately prior to CER, marble burying and locomotor tests. After DHβE injection, animals were returned to their home cages for 15 minutes before CER, elevated plus maze and locomotor testing and for 30 minutes prior to the marble burying task. Weights were measured approximately 1 h prior to behavioral assays.

### Behavioral Procedures

#### Conditioned emotional response (CER)

**Figure 1:**
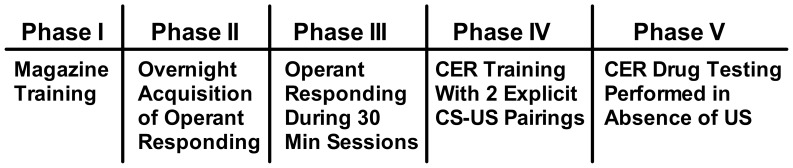
CER training and drug testing schedule. To introduce mice to the location of saccharin delivery, magazine training (Phase I) occurred over days 1–3. CER acquisition of lever pressing maintained by 10 mMol saccharin solution took place during overnight sessions with increasingly demanding schedules of reinforcement (Phase II; days 4–9) until mice reached criteria of 70 reinforcers and 100 s of correct magazine entries in a session. This was followed by daily 30 minute sessions (Phase III) where mice lever pressing was maintained by saccharin under a variable schedule of reinforcement. Mice moved onto the next Phase of training when they reached criteria of 40 lever presses and 10 reinforcers during a single 30 minute session (days 10–13). During CER training mice continued operant training but also received 2 explicit pairings of a light and tone conditioned stimulus (CS) which co-terminated with a 0.1 or 0.3 mA footshock unconditioned stimulus (US) (Phase IV; days 14–43). Phase IV continued until all mice showed a stable level of CS and NON-CS responding over 3 days. Drug testing (Phase V) consisted of lever pressing maintained by saccharin in the presence of the CS but in the absence of the US. For these studies that used a within-subject, Latin square design, there were at least 2 CER training days in between drug testing sessions to assure that mice returned to baseline prior to the next injection of drug.

The CER paradigm consisted of several phases of operant and Pavlovian training followed by drug testing sessions where operant responding was tested in the presence of a footshock-paired CS but in the absence of the US footshock (Figure 1).

#### Magazine training

Subjects received eighty presentations of 10 mMol saccharin solution according to a variable interval 30 second (VI-30 s) schedule. Mice met criteria when they entered the magazine for 20% of the dipper presentations. Animals failing to meet this criteria were given a second, and if necessary, a third exposure to magazine training before moving on to acquisition of operant responding during overnight sessions.

#### CER acquisition of lever-pressing behavior

Mice were trained to lever press for 0.01 ml of 10 mMol saccharin solution delivered via liquid dipper during a single 16 hour overnight session (adapted from [35]). Responding for saccharin reinforcement was maintained on increasing fixed ratio (FR) schedules, FR 1 up to 10 reinforcers, FR 2 up to the subsequent 10 reinforcers and FR 4 for the subsequent 20 reinforcers. This was followed by a variable ratio (VR) 5 or variable interval (VI) 15 second schedule of reinforcement until the end of the session. Mice were next trained to lever press during 30 minute daily sessions (1400–1800 h) for a saccharin reinforcer at the assigned variable schedule of reinforcement for which they were trained. Mice meeting a criterion of 10 reinforcers and 40 lever presses in a single session advanced to CER training. The house light was on during all acquisition and CER procedures in the absence of the CS.

#### CER training (operant responding with pavlovian CS + US footshock conditioning)

During 30 minute CER sessions, saccharin continued to be available according to the variable schedule of reinforcement presented during acquisition. Pseudo-random presentation of two 60 s, compound CSs (house light off + cue light on +70 dB, 2000 Hz tone) co-terminated with a 0.5 s, 0.3 mA footshock US. A second cohort of animals received all the same conditions but was administered a 0.1 mA footshock US shown previously to not affect suppression ratios [36]. The first CS presentation occurred between min 3 and 12 and the second CS between min 18 and 27. The number of lever presses were recorded both for the 60 s Pre-CS period, immediately preceding the onset of the CS, as well as during the CS. The suppression ratio was calculated using the equation A/(A+B), where A is the number of responses during the CS and B equals lever presses during the Pre-CS period [37]. A suppression ratio of 0.5 indicates no suppression of responding during the CS and a suppression ratio of 0 reflects total suppression of responding during the CS. All active lever-pressing in the absence of the CS was also evaluated (NON-CS). Increases in suppression ratio following drug treatment were interpreted as anxiolytic-like behavior. Once stable baseline responding and suppression ratios (≤0.1 for the 0.3 mA condition) were established for 3 consecutive days, mice proceeded to drug testing.

#### CER drug testing

Drug testing took place in the absence of footshock US using a within-subject Latin square design. Animals received 0, 0.0032, 0.01, 0.032 or 0.1 mg/kg i.p. nicotine or 0, 0.1, 0.3, 1 or 3 mg/kg i.p. DHβE before CER. At least 2 days of CER training were administered between doses to allow for wash-out of drug. These intermediate training sessions further assured that responding returned to baseline between doses of drug.

#### Marble burying

Using a within-subject, Latin Square design, separate groups of mice received 0, 0.01 and 0.032 mg/kg i.p. nicotine or 0 and 3 mg/kg i.p. DHβE. Marble burying sessions were separated by at least 5 days as has been demonstrated to provide a steady level of digging behavior in the absence of treatment [38], [39]. Prior to each test, 20 green, glass marbles (10 mm diameter) were evenly arranged in a 4×5 grid on sawdust bedding. Individual mice were placed into the side of the experimental cage so as to not disturb any of the marbles. At the conclusion of the 15 minute test, mice were returned to the home cage; marbles at least 50% covered by the bedding were counted as buried.

#### Elevated plus maze

Using a between-subject design, mice receiving 0, 0.03 or 3 mg/kg i.p. DHβE were returned to their home cage for a 15 min wait period and subsequently placed on the center of an elevated plus maze apparatus (n = 9−11 per dose). Behavior was evaluated for a period of 10 minutes. Subjects were scored for open arm entries, time spent in the open arms and latency to explore the terminal zones (the extreme 5 cm) of the open arms.

#### Locomotor test

Using a between-subject design, animals received nicotine (0, 0.01 or 0.032 mg/kg i.p.) immediately prior to placement into the small chamber of the Med Associates apparatus. The door separating the two chambers was opened, allowing animals free mobility throughout the apparatus. Breaking of two adjacent beams (3 cm equidistant apart) constituted a locomotor activity count. Behavior was assessed for ten minutes. Mice that received DHβE (0, 0.3 or 3 mg/kg i.p.) were placed in their home cages for 15 min following injections with the other locomotor procedures as described for nicotine subjects.

### Statistical Analysis

For CER experiments, repeated measures ANOVA assessed the effect of nicotine and DHβE on suppression ratio, lever presses/minute during the CS period, the Pre-CS period and the NON-CS period. Paired t-tests were used as *post hoc* tests where appropriate. Student’s t-test was used to assess the effect of footshock intensity on suppression ratios. Repeated measures ANOVA and paired t-tests were used to evaluate the effect of nicotine and DHβE on number of marbles buried. ANOVA tests assessed elevated plus maze activity as measured by open arm entries, time on the open arms and latency to reach the end terminus of the open arms. *Post hoc* t-tests and planned comparisons were performed between vehicle- and drug-injected subjects. One-way ANOVA tested the effects of drug doses on locomotor activity. Confidence intervals of *p*<0.05 were reported as significant.

## Results

**Figure 2:**
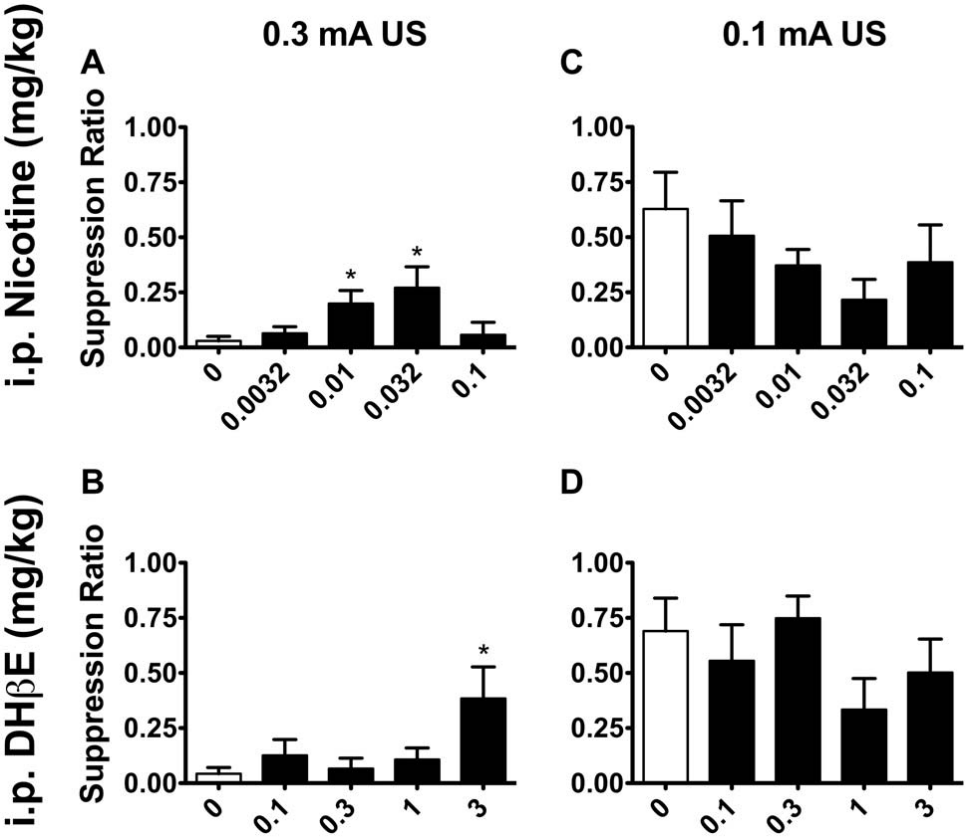
Low dose nicotine and the β2*nAChR antagonist DHβE reverse conditioned suppression. In mice trained to a 0.3 mA unconditioned stimulus footshock during CER training, A) administration of nicotine resulted in a dose-dependent reversal of conditioned suppression as measured by increased suppression ratios (*F*_4,9_ = 3.101, *p*<0.05; n = 10). B) The β2*nAChR antagonist DHβE also resulted in a significant increase in suppression ratios in these mice (*F*_4,6_ = 2.934, *p*<0.05; n = 7), suggesting that inhibition of β2*nAChRs supports anxiolytic-like behavior in the CER assay. C, D) Neither nicotine (*F*_4,5_ = 1.991, *p*>0.05; n = 6) nor DHβE (*F*_4,5_ = 1.263, *p*>0.05; n = 6) resulted in significant changes in suppression ratio responding in mice exposed to 0.1 mA US footshock during CER training. Data are presented as means ± SEM. *p<0.05 compared to when mice received saline control injections.

On the 3 days of CER training prior to CER testing, NON-CS responding was stable (*F* <1) and suppression ratios were consistently lower than 0.1 for mice trained to a 0.3 mA footshock. The suppression ratios for mice receiving 0.1 mA footshock US were significantly higher than mice exposed to 0.3 mA footshock US following saline injection (0.1 mA = 0.69±0.15; 0.3 mA = 0.03±0.02; *t*_14_ = 5.691, *p*<0.001). In contrast to mice that received a 0.3 mA footshock, mice trained with a 0.1 mA footshock did not show a suppression of responding during the CS, indicating that this suppression of behavior in 0.3 mA-trained mice was a conditioned response to an aversive CS and not due to a more generalized orienting response to the compound stimulus CS used in these experiments. There was no effect of footshock intensity on overall NON-CS or Pre-CS lever pressing (*F*’s <1). Figure S1 shows average lever presses per minute during the entire 30 minute sessions. Administration of nicotine resulted in a dose-dependent increase in suppression ratio (*F*_4,9_ = 3.101, *p*<0.05) for mice exposed to the 0.3 mA US. *Post hoc* t-tests revealed that low doses of nicotine (0.01 and 0.032 mg/kg) significantly reversed conditioned suppression of responding in comparison to when animals received saline (*t*_9_ = 2.663 and 2.331, *p*’s <0.05; Fig. 2A). Despite consistent trends for elevated responding at doses of nicotine that reversed conditioned suppression, raw scores for CS lever pressing failed to reach significance following nicotine injection (*F*_4,9_ = 0.867, *p*>0.05; Table 1). Unlike suppression ratios, there was no effect of drug treatment on Pre-CS responding (*F*_4,9_ = 1.771, *p*>0.05). There was an effect of nicotine treatment observed for NON-CS responding, however (*F*_4,9_ = 9.832, *p*<0.001). *Post hoc* tests showed that NON-CS lever pressing was elevated in mice following 0.0032 mg/kg (*t*_9_ = 3.820, *p*<0.01), 0.032 mg/kg (*t*_9_ = 4.941, *p*<0.001) and 0.1 mg/kg (*t*_9_ = 2.483, *p*<0.05) compared to treatment with saline (Table 2). It is possible that nicotine was enhancing the reinforcing efficacy of the saccharin stimulus as has been observed for a visual cue [40], [41]. Further analysis comparing mice against their responding prior to any injections indicated that this may have been due in part to anxiolytic effects of nicotine as well. Compared to days when they had received no injection, there was a significant decrease in NON-CS responding of mice following saline injection (*t*_9_ = 4.683, *p*<0.001), suggesting that the stress of injection led to an overall reduction in lever pressing activity (Table 2). Nicotine injection appeared to reverse this effect; low doses of nicotine (0.01 and 0.032 mg/kg i.p.) that elevated suppression ratio responding resulted in NON-CS lever pressing that did not differ from pre-injection responding. A rewarding-like dose of nicotine (0.1 mg/kg i.p.) [27], [28], [34] resulted in a similar elevation of responding compared to saline injection, however, there were also significantly fewer lever presses during the NON-CS compared to when no injection was given, suggesting that this dose was not effective at reversing suppression of overall responding that was stimulated by the stress of injection.

**Table 1:** Lever pressing activity during the CS and Pre-CS period.

	0.1 mA		0.3 mA	
Nicotine (mg/kg)	Pre-CS	CS	Pre-CS	CS
Pre-Drug	2.83±1.17	1.50±0.26	1.60±0.40	0.30±0.17
0	1.58±0.97	1.50±0.47	0.80±0.40	0.10±0.07
0.0032	1.00±0.51	1.50±0.47	2.07±0.11	0.21±0.12
0.01	2.83±1.38	1.58±0.52	1.90±0.49	0.45±0.16
0.032	4.00±1.48	1.58±0.97	3.86±1.79	0.36±0.14
0.1	1.25±0.69	1.17±0.54	2.25±0.68	0.20±0.20
**DHβE (mg/kg)**	**Pre-CS**	**CS**	**Pre-CS**	**CS**
Pre-Drug	2.75±1.33	3.92±1.48	1.86±0.66	0.14±0.09
0	1.58±0.97	1.50±0.47	1.07±0.55	0.14±0.09
0.1	3.50±1.48	2.25±0.68	1.25±0.36	0.25±0.14
0.3	0.75±0.34	1.83±0.38	1.93±0.63	0.14±0.09
1	2.80±0.88	0.80±0.21	2.36±0.70	0.50±0.29
3	1.75±0.48	1.67±0.69	2.86±1.29	1.71±0.79

Lever presses per minute are depicted for mice during presentation of the 60 s conditioned stimulus (CS) and during the 60 s period prior to CS presentation (Pre-CS). Pre-Drug levels of responding are depicted for the last day of training prior to drug testing sessions (Pre-Drug) and during test sessions following each of five i.p. doses of nicotine or DHβE. Data are represented as means ± SEM.

**Table 2:** Lever pressing activity in the absence of the CS.

	0.1 mA	0.3 mA
Nicotine (mg/kg)	NON-CS	NON-CS
Pre Drug	2.68±0.95	1.92±0.28
0	1.89±0.64	0.94±0.22*
0.0032	1.31±0.48	1.78±0.30^+^
0.01	1.58±0.57	1.40±0.15
0.032	1.90±0.67	2.09±0.27^+^
0.1	1.10±0.42	1.30±0.18*^+^
**DHβE (mg/kg)**	**NON-CS**	**NON-CS**
Pre Drug	3.33±0.98	2.87±0.49
0	1.89±0.64*	1.21±0.24*
0.1	1.98±0.69	2.30±0.41
0.3	2.21±0.87	1.83±0.30
1	2.43±0.72	2.17±0.26
3	1.99±0.50	2.41±0.51

Lever presses per minute are depicted for mice in the absence of the conditioned stimulus (NON-CS) on the day prior to drug testing sessions (Pre-Drug) and during test sessions following each of five i.p. doses of nicotine or DHβE. Data are represented as means ± SEM; *Significantly different from Pre-drug training (*p*<0.05); ^+^Significantly different from test sessions following saline injection (0 mg/kg; *p*<0.05).

In mice exposed to the 0.1 mA footshock US, administration of nicotine did not significantly affect suppression ratios (*F*_4,5_ = 1.991, *p*>0.05; Fig. 2C), CS responding (*F*_4,5_ = 0.103, *p*>0.05), Pre-CS responding (*F*_4,5_ = 2.245, *p*>0.05; Table 1) or NON-CS responding (*F*_4,5_ = 1.46, *p*>0.05; Table 2).

A dose-dependent reversal of conditioned suppression was also observed following treatment with the selective β2*nAChR antagonist DHβE (*F*_4,6_ = 2.934, *p*<0.05). Compared to when they received saline, mice showed a significant increase in suppression ratios following injection of 3 mg/kg i.p. DHβE (*t*_6_ = 2.614, *p*<0.05; Fig. 2B) suggesting that antagonism of the β2*nAChRs, like low dose nicotine, reverses conditioned inhibition of behavior in this task. Pre-treatment with DHβE resulted in a dose-dependent increase in total lever pressing in the presence of the CS (*F*_4,6_ = 3.338, *p*<0.05) reflecting a trend for elevated responding during the CS after administration of 3 mg/kg i.p. DHβE compared to saline (*t*_9_ = 2.049, *p* = 0.086; Table 1). As observed during the nicotine treatment regimen above, Pre-CS responding was not significantly affected by DHβE exposure (*F*_4,6_ = 1.382, *p*>0.05; Table 1), but total NON CS lever pressing was reduced in mice following saline injection compared to the training session that immediately preceded the drug testing phase for DHβE (*t*_6_ = 3.113, *p*<0.05; Table 2). Unlike nicotine, DHβE did not significantly affect responding in the absence of the CS (Table 2). Similarly to nicotine, mice trained with 0.1 mA US footshock showed no effects of i.p. DHβE on suppression ratios (*F*_4,5_ = 1.263, *p*>0.05; Fig. 2D), CS lever pressing (*F*_4,5_ = 1.334, *p*>0.05), Pre-CS responding (*F*_4,5_ = 2.274, *p*>0.05; Table 1) or NON-CS responding (*F*_4,5_ = 1.112, *p*>0.05; Table 2). As observed with 0.3 mA-trained mice, NON-CS responding was lower in mice following saline injection compared to responding during training sessions immediately prior to DHβE drug testing (*t*_5_ = 3.451, *p*<0.05; Table 2), suggesting that the stress of injection may have led to a suppression of overall lever-pressing activity.

**Figure 3:**
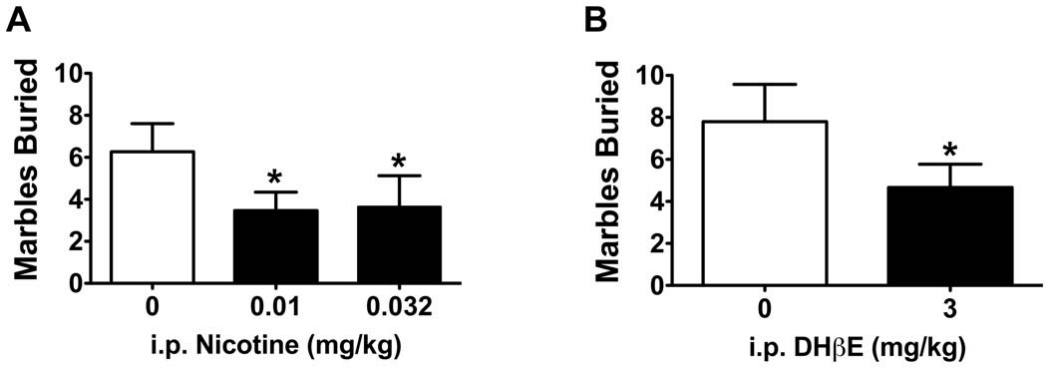
Nicotine and DHβE resulted in fewer marbles buried. A) The 0.01 and 0.032 mg/kg i.p. nicotine that promoted anxiolytic-like behavior in the CER task also resulted in a significant reduction in digging behavior as measured by fewer marbles buried compared to when mice were administered saline (n = 14). B) Similarly, mice treated with 3 mg/kg i.p. DHβE also buried less marbles compared to when they received saline treatment (n = 15). Data are presented as means ± SEM; *p<0.05 compared to when mice received saline control injections.

A separate group of mice were tested in a marble burying task, an ethological measure of digging behavior that is thought to reflect changes in rodent affect [38], [42]–[50]. Doses of i.p. nicotine (0.01 and 0.032 mg/kg) and i.p. DHβE (3 mg/kg) that were capable of increasing lever pressing maintained by saccharin during presentation of an aversive CS also led to a significant decrease of marble burying in an open, exposed environment. Repeated measures ANOVA revealed a significant effect of nicotine exposure on digging behavior as measured by marbles buried (*F*_2,13_ = 4.022, *p*<0.05). Consistent with results from CER, *post hoc* t-tests revealed that mice buried fewer marbles after 0.01 and 0.032 mg/kg i.p. nicotine than when they received saline (*t_13_* = 2.747, *p*<0.05 and *t_13_* = 2.376, *p*<0.05, respectively; Fig. 3A). DHβE-injected mice also buried significantly fewer marbles than after they received saline vehicle in the marble burying task (*t_14_* = 1.781, *p*<0.05; Fig. 3B).

**Figure 4:**
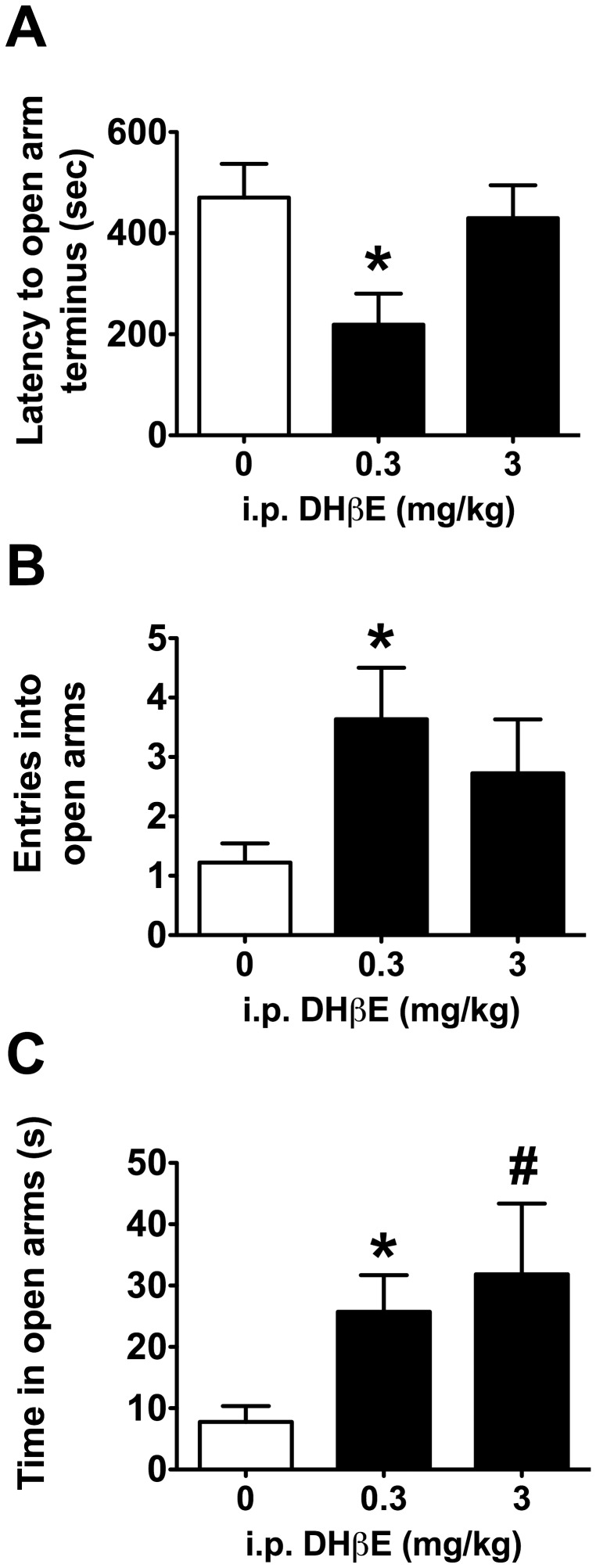
Antagonism of β2*nAChRs promoted anxiolysis-like behavior in the elevated plus maze. A) Mice receiving 0.3 mg/kg i.p. DHβE required less time to explore the end terminus of the open arms of an elevated plus maze, B) made more entries into the open arms of the maze and C) spent more time in the open arms than saline-injected mice. Data are presented as means ± SEM; *p<0.05, #*p* = 0.067 compared to saline controls.

Antagonism of β2*nAChRs also affected the behavior of mice in the elevated plus maze. ANOVA analysis revealed a significant effect of DHβE treatment on latency to reach the terminus of the open arms (*F*_2,30_ = 4.449, *p*<0.05). *Post hoc* tests revealed that mice receiving 0.3 mg/kg i.p. DHβE required significantly less time to explore the terminal ends of the open arms compared to mice receiving saline (*t*_17.341_ = 2.769, p<0.05; Fig. 4A). Despite similar trends for DHβE-associated increases in open arm entries and total time spent in the open arms, ANOVA tests failed to return a significant effect of treatment for these respective measures (*F*_2,30_ = 2.258, *p*>0.05; *F*_2,30_ = 2.219, *p*>0.05; Fig. 4), but planned comparisons revealed that mice receiving 0.3 mg/kg i.p. DHβE had a significantly greater number of open arm entries than saline-injected mice (*t*_12.682_ = 2.610 *p*<0.05; Fig. 4B) and spent significantly more time in the open arms of the maze compared to saline controls (*t*_13.490_ = 2.753, *p*<0.05; Fig. 4C). Although there was a trend for mice receiving 3 mg/kg DHβE to spend more time in the open arms (*t*_10.992_ = 2.034, *p* = 0.068), behavioral measures for this dose of DHβE failed to reach significance for any behavioral measure in the elevated plus maze.

**Figure 5:**
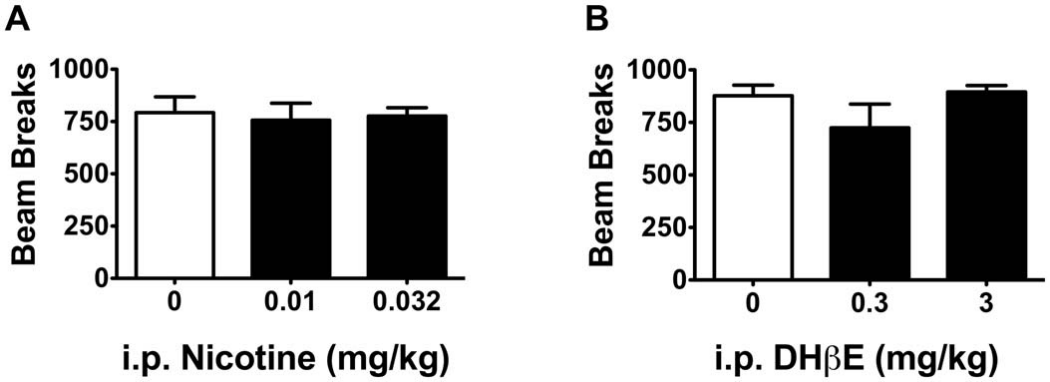
Nicotine and DHβE did not affect locomotor activity. Doses of A) nicotine (n = 5−6) and B) DHβE (n = 4−5 per group) that promoted anxiolytic-like behavior in the CER and marble burying tasks did not affect locomotor activity as measured by beam breaks (*F*_s_ = 0.072, 1.451, *p*_s_>0.05). Data are presented as means ± SEM.

To further determine if the observed behavioral effects of nicotine and DHβE were due in part to non-specific changes in locomotion, mice were tested in a locomotor activity beam-break apparatus following administration of saline and doses of nicotine (0.01 and 0.032 mg/kg) and DHβE (0.3 and 3 mg/kg) that reversed conditioned suppression in the CER assay, that decreased digging in the marble burying task, or that increased open arm activity during the elevated plus maze test. In comparison to saline-injected animals, there were no observable effects of i.p. nicotine (*F*_2,14_ = 0.072, *p*>0.05) or DHβE (*F*_2,13_ = 1.451, *p*>0.05) on locomotor activity (Fig. 5).

## Discussion

In the present experiments, low dose nicotine and a selective antagonist of β2*nAChRs decreased fear- and anxiety-like behavior in three separate animal models of affect. There was a significant reversal of conditioned suppression of lever pressing in mice treated with 0.01 and 0.032 mg/kg i.p. nicotine but a 0.1 mg/kg i.p. dose of nicotine that has been shown to be rewarding during conditioned place preference [27], [28], [34] had no effect. Mice treated with these low doses of nicotine also buried fewer marbles compared to when they were treated with saline in an ethological marble burying task, and previous studies show that similarly low doses of nicotine decrease anxiety-like behavior as measured by increases in open arm activity in an elevated plus maze [27], [51]. The present findings expand on previous data to show that inactivation of the high affinity β2*nAChRs has similar effects of low dose nicotine on these affective tasks. DHβE dose dependently increased responding for a saccharin reinforcer during the presentation of an aversive CS, significantly decreased marble burying in an open, exposed environment and significantly increased exploration of the open arms of an elevated plus maze. These divergent behavior-stimulating and behavior-inhibiting measures indicate that these observations were not due to non-selective effects of DHβE or nicotine on activity. Neither effective doses of nicotine nor DHβE showed any change in beam break activity during a locomotor task. Together these findings suggest that low dose nicotine and DHβE attenuate negative affective and anxiety-like behavior.

Together with previous findings, these studies identify a dual role for β2*nAChRs in regulating nicotine reinforcement and relieving negative affective behavior. Whereas nicotine reinforcement and reward-like behavior require activation of β2*nAChRs [21]–[27], [29]–[32], [52], the studies described herein suggest that inactivation of β2*nAChRs decreases fear-like and anxiety-like behavior as measured by increased suppression ratios during CER, decreased digging behavior in a marble burying task, and increased exploratory behavior in the open arms of an elevated plus maze. Rats will self-administer A-85830, a selective agonist of β2*nAChRs [52]. Administration of selective β2*nAChR antagonists blocks nicotine conditioned place preference and greatly attenuates nicotine self-administration [21]–[25]. This is in contrast to the current studies which show that systemic administration of DHβE promotes lever pressing maintained by saccharin during presentation of a stressful cue. The current studies also showed that subthreshold doses for nicotine conditioned place preference, but not a reward-like dose, were capable of increasing suppression ratios during the CER operant task. The non-selective nAChR antagonist mecamylamine has been shown to have anxiolytic efficacy in the elevated plus maze, social interaction and marble burying tasks [53,54,551,56]. The present findings expand on this work to show that inhibition of β2*nAChRs is sufficient to decrease fear-like behavior and to increase anxiolytic-like behavior in Pavlovian/operant and ethological tasks.

Low dose nicotine had similar effects as DHβE to decrease negative affective behavior. Although the mechanism of how DHβE and nicotine act at nAChRs has not been clearly elucidated in an awake, behaving animal, *in vitro* and *ex vivo* studies show that nicotine promotes both activation and desensitization of nAChRs [17]–[20], [57]–[59]; hence nicotine-associated desensitization could result in a behavioral phenotype that is similar to nAChR antagonism. Micromolar concentrations of nicotine activate β2*nAChRs, facilitating neurotransmitter release [17]–[20]. This is followed by rapid desensitization of the β2*nAChRs [17]–[20]. *In vitro* studies further show that nanomolar concentrations of nicotine can result in preferential desensitization of β2*nAChRs [17], [57]–[59]. These studies observed a more robust reversal of conditioned suppression with DHβE than for low doses of nicotine. This is likely due to the mixed agonist and desensitizing properties of nicotine. Unlike complete inactivation of the receptor as would occur with antagonist binding, at nicotine equilibrium, nAChRs are thought to be “smoldering,” i.e. capable of desensitization and activation, depending strongly on nicotine concentration [57], [60]. Low levels of nicotine increase the likelihood that β2*nAChR stoichiometry will favor the desensitized state. Consistent with these observations, reductions in marble burying are also observed following administration of partial agonists of β2*nAChRs, including varenicline and sazetidine-a [45], [54], [61], [62]. The present data suggest that behavioral effects of partial agonists in the marble burying and CER tasks are likely due to inhibition rather than activation of the β2*nAChRs.

It is not clear from these studies which nAChR subunits in combination with β2 might require inactivation to promote the anxiolytic-like effects of nicotine. DHβE has high affinity for α6β2*nAChRs and α4β2*nAChRs [3], [63] although a large part of the sensitivity appears to be driven by the α4 subunit [63]. Recent work using the elevated plus maze as a measure of anxiety reported that α4 knockout mice fail to show nicotine-associated anxiolysis behavior [27]. There was no genotypic effect of the α4 null mutation in the absence of drug, so it is not clear if activation or inhibition of α4β2*nAChRs or perhaps some other αβ*nAChR is regulating open arm activity in this task [27], [64], [65]. Selective deletion of the α4 subunit in ventral tegmental area (VTA) dopamine (DA) neurons attenuated the effects of nicotine on open arm entries in the elevated plus maze, suggesting a possible role for the mesolimbic DA pathway in support of anxiolysis-like behavior [27]. Given recent data to suggest that VTA GABA neurons promote conditioned aversion and counter appetitive behavior via inhibition of DA neuron signaling [66], [67], it is possible that blockade of α4β2*nAChR activity on GABA terminals could promote anxiolysis-like behavior via disinhibition of DA neurons [19], [20], [28]. It is not clear if α6β2*nAChRs, which are enriched in catecholaminergic nuclei [15], [68], [69] (but see [70]) might contribute to anxiety-like behavior. Slice electrophysiology studies show that α4α6β2*nAChRs on DA neurons in the posterior VTA are highly sensitive to even nM concentrations of nicotine and are resistant to desensitization [71] suggesting that their activity, rather than their inhibition, may promote nicotine-associated anxiolysis in response to low doses of nicotine. As further support that desensitization of α4β2*nAChRs promotes anxiolysis-like behavior, the α4β2*nAChRs, but not α6β2*nAChRs, are localized in the basolateral amygdala where selective removal of ACh inputs decreases anxiety-like behavior [7], [15], [68], [69], [72], [73]. A lack of compounds with selectivity for α6β2*nAChRs that cross the blood brain barrier make it difficult to assess the stoichiometry of the β2*nAChRs that support the systemic effects of nicotine; future studies using selective peptide infusions in brain e.g. [21], [22], [24] will help parse the subunit configurations in combination with β2 that promote anxiolysis via inhibition of β2*nAChRs.

Behaviorally, these nicotine findings are consistent with previous data. A preponderance of the evidence suggests that low doses of nicotine promote anxiolysis-like behavior [27], [74]–[76], moderate doses of nicotine support reward-like behavior [27], [28], [34], and high doses of nicotine increase anxiety-like behaviors [74], [75], [77], [78]. Similarly to low dose nicotine and DHβE, anxiolytic drugs such as benzodiazepines increase lever pressing during a presentation of an aversive CS compared to when saline is administered [35]–[37], [79]–[83], decrease digging in the marble burying task [38], [42]–[45] and increase open arm activity in an elevated plus maze. Studies in humans show that trait anxiety leads to elevated cued fear conditioning of aversive stimuli and imaging studies show this behavioral tendency is positively correlated with an exaggerated activation of the amygdala and anterior cingulate cortex, brain areas shown to regulate rodent behavior during fear conditioning tasks [84]–[89]. CER, marble burying and the elevated plus maze have good predictive validity for anxiolytic drug efficacy [35]–[38], [42]–[47], [79]–[83], [90]–[94]. Together with previous data, the present studies suggest that inactivation of nAChRs may promote anxiolysis-like behavior and may have mechanistic implications for why individuals smoke to relieve anxiety.

These studies utilized CER, marble-burying and an elevated plus maze task to show that nicotine and DHβE could both stimulate and suppress behavior in a way that is consistent with currently available anxiolytic drugs [38], [43]–[47], [90]–[94]. Marble burying, however is also sensitive to antidepressant drugs and antipsychotics [44], [48]–[50] suggesting that digging behavior in rodents may be driven by an underlying system that is common to the effects of these diverse drug classes. Individuals diagnosed with anxiety disorder, depression or schizophrenia all have a significantly elevated risk for tobacco dependence [95], [96]. In addition to the high concordance with tobacco use, there is a high comorbidity for diagnosis of anxiety with depression and schizophrenia, suggesting that there is a common underlying etiology for these disorders [97]. Some suggest that the “non-purposeful” digging behavior in the marble burying task may model obsessive compulsive anxiety disorder [48]–[50]. Drugs such as clozapine, apiprizole and risperidone that are used to augment the effects of mood stabilizers also reduce marble burying activity [48], [49]. β2*nAChRs are ubiquitously expressed in the brain [15], [68], [69], [98], [99] where their activation on the neuron soma and terminals promotes release of GABA, serotonin, dopamine, norepinephrine and acetylcholine, neurotransmitters that regulate mood and arousal and that are believed to contribute to the etiology of anxiety, depression and schizophrenia [100]. The β2*nAChRs have also been implicated in contributing to rodent models of depression-like behavior with mecamylamine and partial agonists of β2*nAChRs showing anti-depressant-like efficacy [101]–[103]. Unlike our observations in the marble burying task, however, administration of DHβE blocks the antidepressant-like effects of the β2*nAChR partial agonists varenicline and sazetidine in the forced swim task [104], showing a dichotomy with the present results in the elevated plus maze which suggest that antagonism of β2*nAChRs promotes anxiolysis-like behavior.

It is possible that our findings in the CER task reflect changes in learning that are independent of fear and anxiety-like behavior. While it is possible that drug injection could result in state-dependent learning effects, we do not believe this was the case given that animals showed dose-dependent effects of nicotine and DHβE using a within-subject, Latin Square design. Place conditioning and drug discrimination studies clearly demonstrate that mice can physiologically detect the 0.1 mg/kg dose of nicotine [27], [28], [34], [105]–[108] yet this dose did not reverse conditioned suppression as low doses did in these studies, suggesting that the effects of nicotine and DHβE on suppression ratios during CER were not due to a generalized decrement caused by state-dependent learning. It is also possible that the injection itself could have served as an occasion-setter to indicate that no shock would occur during these test sessions. This was not the case. Rather, saline injection led to a decrease in NON-CS responding during these test sessions, suggesting that the stress of injection led to a reduction in goal-oriented behavior as measured by lever pressing for saccharin. Several doses of nicotine, including a rewarding-like dose, reversed this suppression of NON-CS responding. Whereas it is possible that this behavior was stimulated by anxiolytic-like effects of nicotine, it is equally plausible that nicotine exposure promoted stimulus enhancing effects of the saccharin reinforcer as has been shown for an unconditioned stimulus light and a conditioned stimulus associated with an appetitive stimulus [40], [41], [109], [110]. The present results also showed an interesting contrast to findings using Pavlovian fear conditioning without an operant component. Unlike our observations in the CER task, systemic administration of nicotine enhances freezing in a footshock-paired context with no effect on explicit cue conditioning [111], [112]. These dichotomies may be due in part to the use of a more mild footshock and extended explicit cue CS training used during CER compared to traditional Pavlovian fear conditioning procedures. A significant difference in CS but not NON-CS lever pressing between mice trained to a 0.1 mA and 0.3 mA footshock suggests that the contextual fear did not contribute to CER behavior in these studies. In addition, systemic administration of DHβE alone does not affect either context or explicit cue CS-freezing following fear conditioning [113], drawing a further contrast between these procedures. Together these findings suggest that basic Pavlovian fear conditioning and CER are modeling different behaviors. These data further suggest that CER, but not Pavlovian fear conditioning, is sensitive to inactivation of the high affinity β2*nAChRs. Whereas the CER paradigm is a complex animal model that involves fear learning and operant behavior, this procedure benefits from subjects acting as their own controls both within and between sessions. The fact that mice showed similar effects in the marble-burying task and elevated plus maze, which do not have a learning components to them, supports the hypothesis that affective behavior was modified by nicotine and DHβE during CER.

Studies in human smokers reveal that multiple factors contribute to tobacco use; as well as the pleasure received from smoking, many report that they use tobacco to relieve anxiety or to relax [114]–[120]. The first cigarette of the day results in an abrupt increase in nicotine plasma concentrations that smokers associate with the rewarding effects of the drug [121]–[123]. The nicotine ingested from a single cigarette is sufficient to occupy 80% of β2*nAChRs [124]. During subsequent smoking episodes, smokers achieve smaller increases in nicotine that ought to preferentially favor desensitization of nAChRs [121], [122] if slice electrophysiology, Xenopus oocyte, tissue culture and synaptosome studies are predictive of how nAChRs function *in vivo* [17]–[20], [57]–[59]. Nicotine reaches daily steady-state concentrations in the brains of human smokers between 200–400 nM. ACh is a major neuromodulator in brain that is thought to regulate anxiety-like behavior [73], [125]. As nicotine levels drop, populations of β2*nAChRs in brain regions that regulate anxiety become available for activation by ACh in response to stressful stimuli such as cigarette/tobacco cues [126]–[129]. Hence, in addition to smoking to activate their β2*nAChRs, tobacco users may also be titrating ACh signals via desensitization of the β2*nAChRs, particularly after the first cigarette of the day. Human imaging studies suggest that β2*nAChRs may be critical for nicotine’s ability to curb anxiety in smokers [130], [131] but presently available compounds that assess β2*nAChR occupancy in humans cannot differentiate between receptors in the activated or desensitized state.

To conclude, low dose nicotine and DHβE had similar effects on affective behavior in the CER, marble burying and elevated plus maze tasks. These studies support the hypothesis that nicotine may reduce negative affect and anxiety via desensitization of the high affinity β2*nAChRs. These data further suggest that antagonism of β2*nAChRs may be an effective strategy for promoting tobacco cessation or for relieving anxiety in non-tobacco users.

## Supporting Information

Figure S1 **Operant responding during individual sessions in absence of drug.** Variability in Pre-CS responding was observed in mice exposed to both 0.1 and 0.3 mA footshock unconditioned stimulus (US) over within-subject delivery of both nicotine and DHβE. The timing of the conditioned stimulus (CS) may have contributed to this variability, as operant responding fluctuated within individual sessions.
(DOCX)
