# Supplementary figures and images for: Low Dose Nicotine and Antagonism of β2 Subunit Containing Nicotinic Acetylcholine Receptors Have Similar Effects on Affective Behavior in Mice
Source: PLoS One. 2012 Nov 7;7(11):e48665. doi: 10.1371/journal.pone.0048665 (PMC3492489; doi:10.1371/journal.pone.0048665)

**
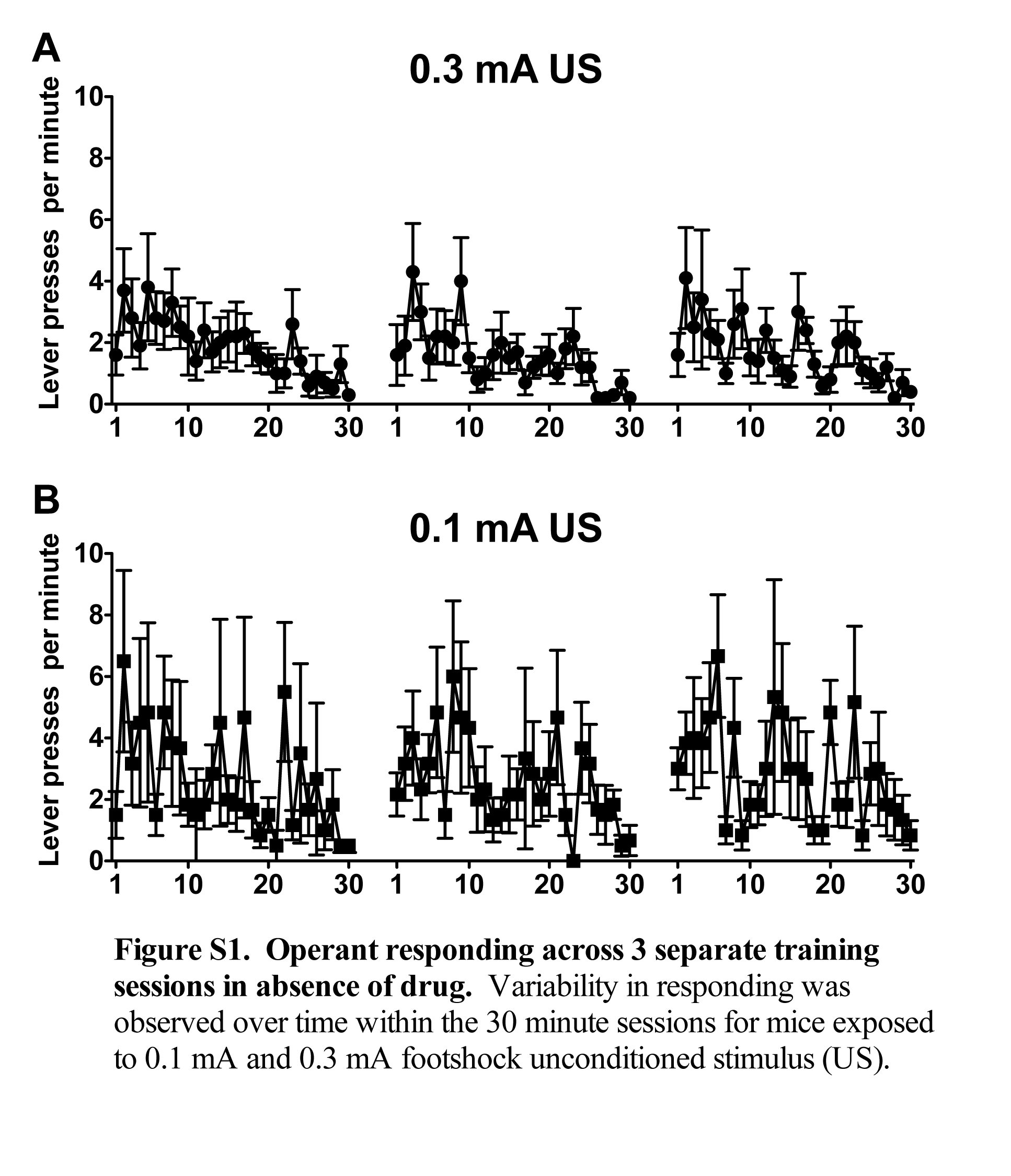
**

Supplement: Figure S1 — Operant responding during individual sessions in absence of drug. Variability in Pre-CS responding was observed in mice exposed to both 0.1 and 0.3 mA footshock unconditioned stimulus (US) over within-subject delivery of both nicotine and DHβE. The timing of the conditioned stimulus (CS) may have contributed to this variability, as operant responding fluctuated within individual sessions. (DOCX) [file pone.0048665.s001.docx]
